# On the Prognostic Significance of Secondary Polycythemia in Cardiopulmonary Affections

**Published:** 1913-11

**Authors:** 


ON THE PROGNOSTIC SIGNIFICANCE OF SECONDARY POLYCYTHEMIA IN CARDIOPULMONARY AFFECTIONS. F. Parkes Weber. London, *Arch. des Mal. du Coeur, des Vaisseaux, et du Sang.*, 1913, VI, 266. The frequent occurrence of polycythemia in congenital heart disease which is accompanied by cyanosis is well known. Few such patients reach adult age. In such patients. however, Weber considers the increased number of red cells to be a compensatory phenomenon; the bone marrow endeavoring to remedy an imperfect oxygenation of the blood by an increased number of erythrocytes. It is possible to find a polycythemia almost as well marked as that seen in congenital cardiac affections in certain patients who have reached the last stages of pulmonary emphysema, chronic bronchitis, asthma, bilateral pleural adhesions, chronic interstitial pneumonia and pulmonary sclerosis of tuberculosis origin. In such cases the occurrence of cyanosis and a pronounced degree of polycythemia is a prognostic sign of considerable gravity. Eleven cases are reported.

